# Effects of Varying Particle Sizes and Different Types of LDH-Modified Anthracite in Simulated Test Columns for Phosphorous Removal

**DOI:** 10.3390/ijerph120606788

**Published:** 2015-06-16

**Authors:** Xiangling Zhang, Qiaozhen Chen, Lu Guo, Hualing Huang, Chongying Ruan

**Affiliations:** School of Civil Engineering and Architecture, Wuhan University of Technology, Wuhan 430070, China; E-Mails: cqz@whut.edu.cn (Q.C.); guolu12_whut@126.com (L.G.); hhlwhut@163.com (H.H.); ruancyvivi@126.com (C.R.)

**Keywords:** LDHs, coating modification, anthracite substrate, phosphorous removal, sewage treatment

## Abstract

A comparative study was carried out for the removal of phosphorus in simulated unplanted vertical-flow constructed wetlands with different layered double hydroxide (LDHs) coated anthracite substrates. Three particle sizes of anthracites were selected and modified separately with nine kinds of LDH coating. The simulated substrates test columns loaded with the original and modified anthracites were constructed to treat the contaminated water. For the medium and large particle size modified anthracite substrates, the purification effects of total phosphorus, total dissolved phosphorus and phosphate were improved by various degrees, and the purification effect of the medium particle size anthracite is better than that of the large size one. The medium size anthracite modified by ZnCo-LDHs had optimal performance with average removal efficiencies of total phosphorus, total dissolved phosphorus and phosphate reaching 95%, 95% and 98%, respectively. The maximum adsorption capacity on ZnCo-LDHs and ZnAl-LDHs modified medium sizes anthracites were 65.79 (mg/kg) and 48.78 (mg/kg), respectively. In comparison, the small size anthracite is not suitable for LDHs modification.

## 1. Introduction

Constructed wetlands are engineered wastewater purification systems that encompass biological, chemical and physical processes [1,2]. Constructed wetlands as a new sewage treatment process are low cost system, easily operated and maintained, and they can be potentially applied in developing countries with serious water pollution problems [3,4]. Substrate is an essential part of vertical-flow constructed wetland system, and it plays an important role in purification of pollutants, especially in phosphorus removal. Meanwhile, it could be the factor which is the most amenable to control [5]. Since different substrates have different sewage purification effects, it is important to select a substrate with a high removal capacity to obtain an efficient purification. Various natural substrates and artificial products have been tested for phosphorus removal such as limestone, zeolite, fly ash, blast furnace slag and anthracite. However, much of these materials were not found to be satisfactory in terms of achieving good adsorption capacity and efficient purification.

With the development of material science, a large number of new functional materials appear, making modification of the natural substrate possible. Layered double hydroxides (LDHs), also known as hydrotalcite or anionic clays, consist of positively charged, brucite-like octahedral layers and a negatively charged interlayer region containing anions and water molecules [6,7]. The general formula of LDHs is $\left[M_{1-x}^{2}+M_{x}^{3+}{\left(OH\right)}_{2}\right]{\left[A^{n-}\right]}_{x/n}\cdot yH_{2}O$, where M^2+^ and M^3+^ are divalent and trivalent metal cations, A^n−^ is the incorporated anions in the interlayer space along with water molecules for charge neutrality and structure stability, and x is defined as the M^3+^/(M^2+^ + M^3+^) ratio with various values between 0.2 to 0.33 [8]. The wide range of possible compositions of LDHs means that materials with a great variety of different properties can be produced [9]. Due to their special properties (acid-basic properties, structure memory effect, interlayer anion exchange capacity and micropore structure) [10,11,12], LDHs have been identified and proposed as adsorbents and ion-exchangers for water treatment.

In recent years, a series of LDHs with different metal cations were synthesized to remove phosphate. Cheng [13] used Zn-Al LDHs to remove phosphate in sewage sludge filtrate. Triantafylidis [14] had synthesized Fe (III)-modified hydrotalcite-like materials (with varying degree of aluminum substitution by iron) as sorbents for phosphate removal. Ashekuzzaman [15] investigated the performance and the potential of the reuse of Ca-, Mg- and CaMg-based LDHs for phosphate removal. In another study [16], Zr^4+^ incorporated MgAl-LDHs with different molar ratios of Mg/(Al + Zr), which were prepared to assess their uptake behavior toward phosphate ions. However, due to appearance structure and small size, LDHs are not suitable for directly application in large-scale sewage treatment. Hence, there is no report yet on the research of LDHs used in constructed wetlands, and there are few reports on the research of LDH-modified substrate.

Herein, we selected three kinds of anthracite including small, medium and large particle size, and utilized three kinds of divalent metal compounds and three kinds of trivalent metal compounds to synthesize LDHs. There were 10 test columns filled with different kinds of coated anthracite. The purpose of this experiment is to investigate the effects of the different LDHs on purifying efficiency, and to provide a reference for controlling and strengthening wetlands purification effect.

## 2. Materials and Methods

### 2.1. Materials

The experimental wastewater was prepared by mixing the raw influent from Long-Wang-Zui sewage treatment plant and waters of South Lake at Wuhan, China. Table 1 summarizes the characteristics of the wastewater used.

**Table 1:** Characteristics of the experiment wastewater (mg/L).

Parameters	TP	TDP	Phosphate
Concentration	0.94~3.91	0.33~2.29	0.30~1.94
Average	2.06	1.37	0.98
Standard deviation	0.73	0.49	0.44

The spherical granular anthracites used in this study were collected from Zhengzhou city, Henan, China. The diameters of small, medium and large size of anthracites are 0.5–1.0 mm, 1.0–3.0 mm, 3.0–5.0 mm, respectively. The chemical analysis of original anthracite is given in Table 2, where it can be seen that anthracite is primarily composed of C, Si and Al oxides. Other minor elements such as Fe, Ca and S are also present. Medium size anthracite has the highest concentration of Al, Fe and Ca oxides.

**Table 2:** The main chemical compositions of original anthracites (%).

Chemical Compositions	SiO_2_	Al_2_O_3_	Fe_2_O_3_	CaO	SO_3_	CO_3_
Percentage Composition (Small Size)	12.10	10.14	2.12	0.73	4.60	68.47
Percentage Composition (Medium Size)	19.35	14.21	4.26	1.78	0.75	53.14
Percentage Composition (Large Size)	16.54	13.46	2.72	0.99	4.32	60.07

**Figure 1:**
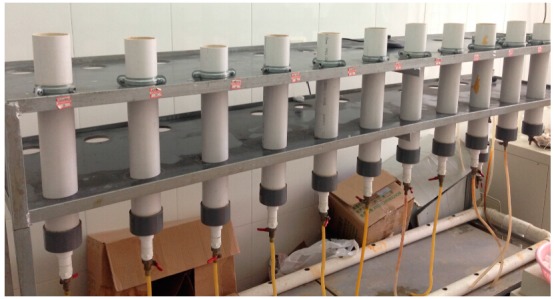
Experimental set-up.

There were 30 experimental columns, ten in each of the three particle sizes of anthracites. The experimental columns were made of polyvinylchloride pipes (PVC) of 0.4 m in length and 0.08 m in diameter. Each column was filled with original or modified anthracites substrates, with a 0.35 m thick layer. Wastewater flowed into the system from the top and out from the bottom. Under the intermittent inflow conditions, the hydraulic retention time of the experiment was 12 h and about 1155 mL of water in each column. The hydraulic loading rate was 230 mm/d. The porosities of the small, medium and lager size anthracites system were 27%, 37% and 40%, respectively. Treatment systems were operated over seven months, from February to October 2013. Each particle size of anthracite was carried out over more than 10 rounds of purification experiments. Samples of influent and effluent from the systems were collected and analyzed in every round for total phosphorus (TP), total dissolved phosphorus (TDP) and phosphate. Figure 1 shows the experimental set-up in the laboratory.

Standard ammonium molybdate method was used. Statistical analysis was performed using the Statistical Product and Service Solutions (SPSS) program (Version 22.0). The level of statistical significance was established at *p* < 0.05 in all cases.

### 2.2. Methods of Coating Modification

Six solutions, 0.2 moL·L^−1^ of CaCl_2_, ZnCl_2_, MgCl_2_ and 0.1 moL·L^−1^ of FeCl_3_, AlCl_3_, CoCl_3_ were prepared and mixed with each other to compose various LDHs. Original anthracites with different particle size ranges were selected, washed and placed into 2 L beakers full of distilled water. The water was heated to 80 °C and kept at this temperature. CaCl_2_ and FeCl_3_ solutions were added into the beaker simultaneously, and 10% of NaOH solution was then added in order to maintain the pH at 11. After vigorous stirring for 4 h, the mixed substrates were centrifuged for 10 min at a speed of 1000–1500 r·min^−1^ and the sediment was then washed to obtain a neutral pH, then dried for 16 h. Finally, the modified anthracites coated with a film of LDHs were obtained. There were nine kinds of LDH modified anthracites obtained in this experiment, and each of the modified anthracites were put into one test column correspondingly, as presented in Table 3.

**Table 3:** Modification methods.

Modification Methods
FeCl_3_ + CaCl_2_ + Anthracites
FeCl_3_ + ZnCl_2_ + Anthracites
FeCl_3_ + MgCl_2_ + Anthracites
CoCl_3_ + CaCl_2_ + Anthracites
CoCl_3_ + ZnCl_2_ + Anthracites
CoCl_3_ + MgCl_2_ + Anthracites
AlCl_3_ + CaCl_2_ + Anthracites
AlCl_3_ + ZnCl_2_ + Anthracites
AlCl_3_ + MgCl_2_ + Anthracites
Original Anthracites

### 2.3. Property Analysis Methods of Modified Anthracites Coated with LDHs

The chemical compositions of three sizes of original anthracites were analyzed by Axios Advanced X-ray fluorescence spectrometer (AXIOS, PANalytical. B. V, Almelo, The Netherlands). The surface morphology and microstructures of the substrates were analyzed by scanning electron microscope (SEM) (Ultra Plus-43-13 SEM, Zeiss, Germany) in Material Research and Testing Center, Wuhan University of Technology.

### 2.4. Adsorption Isotherms Experiment

Medium size modified anthracites were used in adsorption isotherms experiment. For the adsorption of phosphate, a standard solution (400 mg/L) was prepared by KH_2_PO_4_ (GR grade). The adsorption experiments were carried out in 250 mL conical flasks by adding 10 g of substrates into 100 mL KH_2_PO_4_ solutions. Varying phosphorus concentrations ranging from 0 to 32 mg/L (*i.e.*, 0, 1.0, 2.0, 4.0, 8.0, 16.0 and 32.0 mg/L). The conical flasks were shaken for 24 h at 120 revolutions per minute (rpm) at 25 ± 1 °C. The solid adsorbent was separated from the mixture by filtration. The P uptakes were calculated from the decreases in phosphate concentration with reference to those of initial concentrations in solution.

The study data were used to plot linearly transformed Langmuir (1) and Freundlich (2) adsorption equation:

(1) $$\frac{1}{\Gamma }=\frac{1}{\Gamma^{0}}+\frac{A}{\Gamma^{0}}•\frac{1}{c}$$

(2) $$\mathrm{lg}\Gamma =\mathrm{lg}K+\frac{1}{n}\mathrm{lg}c$$

where, *Γ (mg/kg)* is the equilibrium phosphorus concentration on the adsorbent, *c* *(mg/L)* is the equilibrium phosphate concentration in solution, *Γ (mg/kg)* is the maximum monolayer phosphorus adsorption capacity of the adsorbent, and *A* is the Langmuir adsorption equilibrium constant. The constant, *n,* indicates the Freundlich isotherm curvature and *K* is the Freundlich equilibrium constant.

## 3. Results and Discussion

### 3.1. Apparent Characteristics of Anthracite before and after Modification

**Figure 2:**
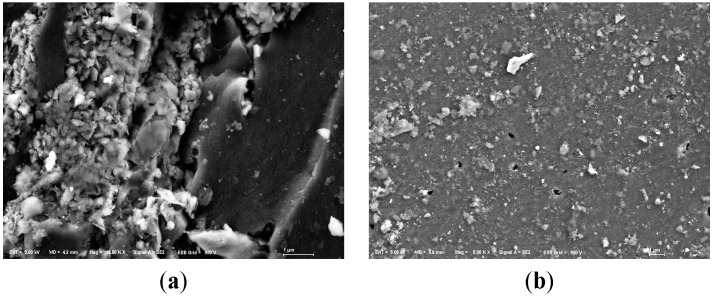

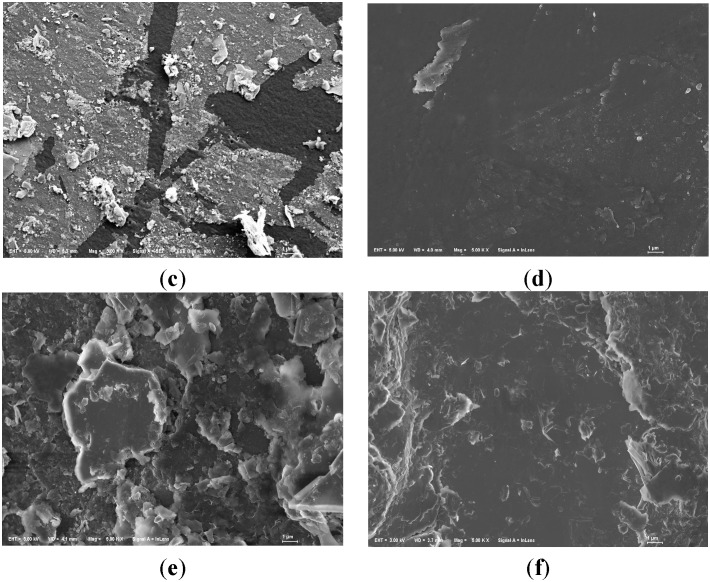
SEM images of anthracite before and after modification. **(a**) Small anthracite coated by ZnAl-LDHs (Mag = ×10,000, Scale: 1 μm); (**b**) Small original anthracite (Mag = ×5000, Scale: 1 μm); (**c**) Medium anthracite coated by ZnCo-LDHs (Mag = ×5000, Scale: 1 μm); (**d**) Medium original anthracite (Mag = ×5000, Scale: 1 μm); (**e**) Large anthracite coated by AlMg-LDHs (Mag = ×5000, Scale: 1 μm); (**f**) Large original anthracite (Mag = ×5000, Scale: 1 μm).

All samples were analyzed by SEM. The SEM images were shown in Figure 2, which just shows original and one of modified anthracites for each particle size, for example. The SEM images for original anthracite revealed the characteristic morphologies of a smooth surface. The SEM images for modified anthracite revealed the characteristic morphologies of a porous surface. Since the modified substrates surfaces were densely covered with a certain amount of white particles, the coated effect was obvious. Different modifying ways shaped different structures of LDHs.

### 3.2. Adsorption Isotherms

The values of isotherm constants are presented in Table 4. The *R*^2^ obtained for the Freundlich and Langmuir isotherms were all above 0.90. In the Freundlich isotherm, the constant *1/n* is a measure of exchange intensity or surface heterogeneity. High values of *1/n* indicate a strong bond between the substrate and the phosphorus. The obtained *1/n* values for all medium size modified anthracite were higher than that for the original anthracite. Larger *K* indicates greater overall adsorption capacity. Moreover, *K* values based on the values of *Γ*^0^ calculated by the Langmuir equation also show the higher capacity by ZnCo-LDHs and ZnAl-LDHs. The maximum adsorption capacity on ZnCo-LDHs and ZnAl-LDHs was 65.79 *(mg/kg)* and 48.78 *(mg/kg)*, respectively.

**Table 4:** The constants and correlation coefficients of the Freundlich and Langmuir Equations for adsorption of phosphate on original and modified anthracites.

Adsorbent (Medium Size Anthracite)	Freundlich Equation			Langmuir Equation		
	1/n	lgK	R^2^	Γ^0^	A	R^2^
CaFe-LDHs	0.612	0.923	0.911	43.10	4.142	0.956
ZnFe-LDHs	0.314	1.407	0.918	40.00	0.172	0.906
MgFe-LDHs	0.351	1.231	0.975	37.31	0.672	0.973
CaCo-LDHs	0.489	1.262	0.900	44.64	0.955	0.932
ZnCo-LDHs	0.347	1.733	0.916	65.79	0.059	0.958
MgCo-LDHs	0.283	1.052	0.996	20.79	0.482	0.908
CaAl-LDHs	0.278	1.220	0.904	32.47	0.526	0.987
ZnAl-LDHs	0.238	1.516	0.978	48.78	0.102	0.969
MgAl-LDHs	0.265	1.449	0.956	43.67	0.135	0.976
original anthracites	0.198	1.488	0.916	39.53	0.0316	0.956

### 3.3. Removal of Phosphorus Pollutants

#### 3.3.1. Removal of Total Phosphorus

**Figure 3:**
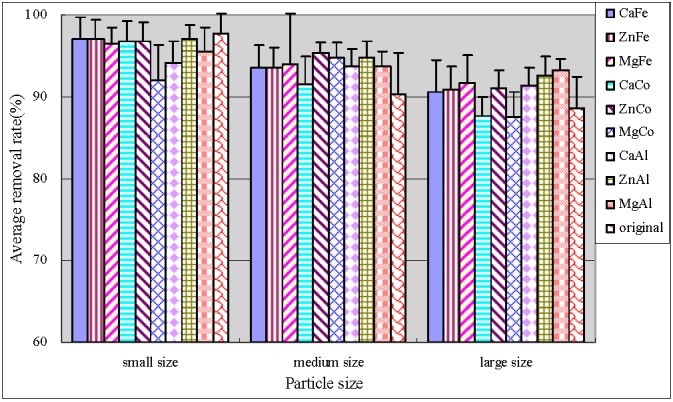
Average removal rate of TP.

**Figure 4:**
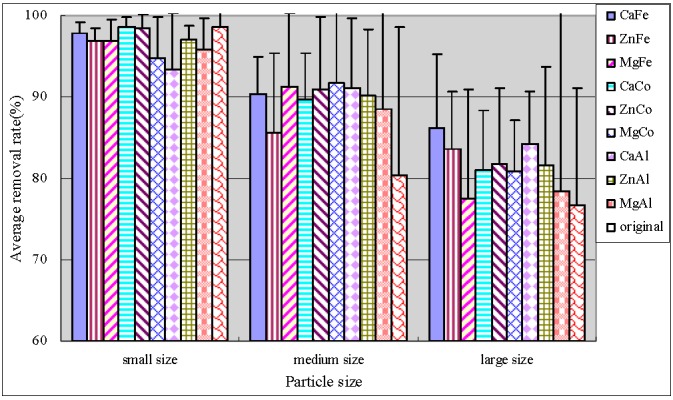
Average removal rate of PP.

Figure 3 and Figure 4 showed the total phosphorus (TP) and particulate phosphorus (PP) average removal rates, respectively. PP was calculated by subtracting the determined TDP values from TP. As can be seen, small original anthracite was most efficient in TP and PP removal with the average removals reaching up to 97% and 90%, respectively. The results of significance test were shown by Table 5. Both small and large size modified anthracite systems showed significantly greater removal efficiencies for TP (*p* < 0.05), and no significant difference in medium size anthracite systems (*p* = 0.248). In addition, average percentage TP and PP removal in both medium and large particle size systems were improved by modification. The medium size anthracite coated by ZnCo-LDHs and by ZnAl-LDHs had optimal performance with average TP removal efficiencies of more than 94.8%. The error bars of TP showed that the treatment behaviors were stable. Zhang *et al.* [17] studied anthracite covered with MgFe-LDHs. They found that both natural and coated anthracite were effective for TP removal. The removal rate was 85% and 92%, respectively.

**Table 5:** ANOVA analysis of removal rates of total phosphorus (TP), particulate phosphorus (PP), total dissolved phosphorus (TDP) and phosphate in the anthracite columns with different coated ways.

Size	TP	PP	TDP	Phosphate
Small	0.000 ***	0.215	0.372	0.044 *
Medium	0.248	0.740	0.096	0.024 *
Large	0.007 ***	0.000 ***	0.000 ***	0.008 ***

The data is *p* values. Significant differences between the original and modified anthracites were analyzed by one-way analysis of variance (ANOVA) method: * *p* < 0.05; *** *p* < 0.001.

The removal mechanisms of TP in constructed wetlands have been identified, for instance not only assimilation and release by vegetation and micro-organisms but also substrate sorption and precipitation [18]. Sorption and precipitation within substrates play the most important role in P removal process, which occurs through physical and chemical ways (such as adsorption, filtration, ion exchange and complexation reaction) [19,20]. The capacity of P-removal was affected by particle size. TP adsorption capacity almost decreased with increasing particle size [21]. With larger specific surface area, small size substrates can widely contact with TP in the wastewater, and can effectively intercept particulate phosphorus. Therefore, small substrates generally had a prominent effect on P removal . However, it cannot secure a high hydraulic conductivity [22]. In addition, the capacity of constructed wetlands to remove P was also dependent on the contents of Ca, Mg, Fe and Al in the substrate. Phosphorus was removed by reacting with Ca, Mg, Fe and Al in the sewage water. It may also be bound in the substrates as a consequence of physical and chemical adsorption and precipitation reactions with Ca, Mg, Fe and Al contained in the substrates [23,24,25]. Our data showed that the medium modified anthracite systems were highly effective and steady in removing TP. This is mainly attributed to the porous microstructures of LDHs as well as their characteristics. With the porous structure, substrates can intercept and adsorb particulate phosphorus effectively.

#### 3.3.2. Removal of Total Dissolved Phosphorus

**Figure 5:**
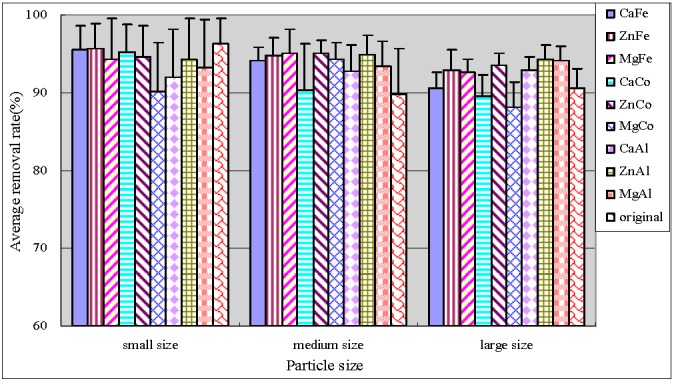
Average removal rate of TDP.

Figure 5 illustrated that small original anthracite had optimal performance and the removal rate of the middle modified anthracite was obviously improved. The medium size anthracites coated by ZnCo-LDHs and ZnAl-LDHs had optimal performance with average TDP removal efficiencies of more than 95%, and the removal effects were stable. Statistics were performed using SPSS software. It was found that there were significant differences between the average TDP removal rates of original and modified anthracites in the large anthracite system (Table 5).

Since TDP is soluble in wastewater, it is difficult to achieve a certain effect using a simple physical filter. The processing mechanisms of TDP mainly focused on chemical adsorption, chemical reactions and biological treatment [26]. Due to the formation of the porous microstructures and the increase of metal ions, modified anthracites enhance the adsorption ability and improve the effect of microbial biodegradation by formation of biofilms, and the TDP-removal rates were improved in the medium and large modified systems.

#### 3.3.3. Removal of Phosphate

**Figure 6:**
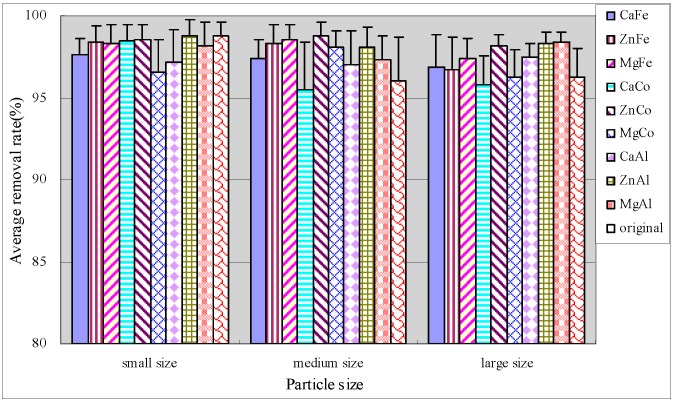
Average removal rate of phosphate.

Anthracite was also efficient for phosphate removal with an average removal reaching up to 95% (Figure 6). In medium size and large size anthracite systems, modification slightly improved the removal rates of phosphate and the stabilities of purification effect as compared to the original anthracite. The behavior of all modified substrate systems in terms of phosphate removal differed significantly (*p* < 0.05), indicating the modification effect on phosphate removal. For constructed wetlands, the phosphate removal mainly relied on chemical adsorption and precipitation reactions. The metal ions of substrate had a good effect on removal of phosphate. When the substrate contained much iron and lead oxides, phosphate would be adsorbed to their surface by ligand exchange; while in substrates with high calcium content, phosphate will react with calcium ions to form insoluble calcium phosphate, which can be removed by precipitation [27].

According to the treatment effects of various forms of phosphorus, the removal mechanism of phosphorus is physical interception, adsorption, chemical reaction and biological reaction in small anthracite systems, and chemical reaction and biological reactions dominate in medium and large anthracite systems. For example, the medium size and large size anthracite coated by MgFe-LDHs and MgAl-LDHs performed well with average removal efficiencies of TP and TDP, and had weak performances with PP removal efficiencies, which show that chemical reaction plays the most important role in phosphorus purification for modified anthracites. In large anthracite systems, the average removal rate of PP of MgFe-LDHs, MgAl-LDHs and original is similar, but the removal effects of TDP of modified anthracites are better than that of original substrates, so the TP-removal rates of the former are much higher. Therefore, anthracites coated by MgFe-LDHs and MgAl-LDHs can improve their chemical reaction ability.

**Figure 7:**
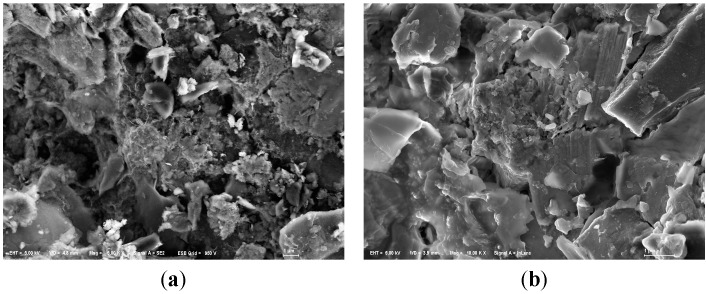

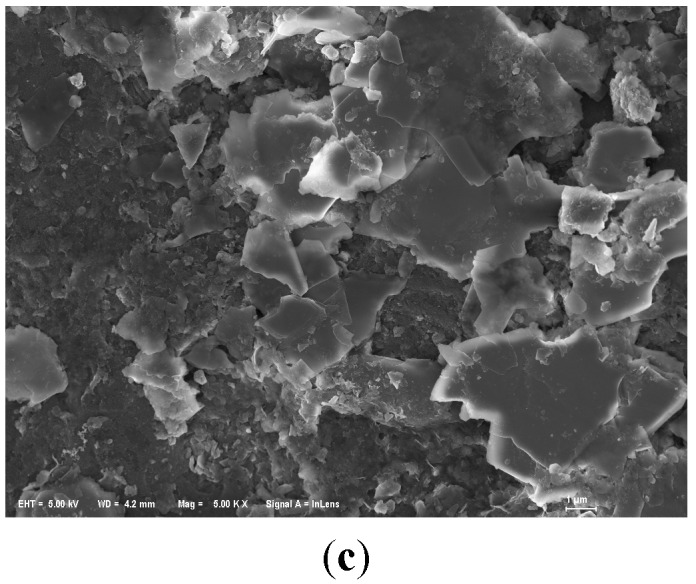
SEM images of modified anthracite after purification experiments. (**a**) Small used anthracite coated by ZnAl-LDHs (Mag = ×5000, Scale: 1 μm); (**b**) Medium used anthracite coated by ZnCo-LDHs (Mag = ×10,000, Scale: 1 μm); (**c**) Large used anthracite coated by MgAl-LDHs (Mag = ×5000, Scale: 1 μm).

On the other hand, we could observe that the substrates were covered with sticky substances during the purification experiments. But in SEM images of used anthracite (after purification experiment) (Figure 7), there was not much significant biofilm, indicating that microorganisms may have a modest effect in phosphorus removal.

## 4. Conclusions

Small particle size of original anthracite performed best for phosphorus removal. After modification, the purification effects of phosphorus in small anthracite systems declined by various degrees. The results suggest that small size anthracite is not suitable for LDH modification.

After modification, the medium particle size anthracite coated by ZnCo-LDHs and ZnAl-LDHs had an average removal efficiency of TP, TDP and phosphate, reaching 95%, 95% and 98%, respectively. These results show that medium and large size anthracites are suitable for coating modification. Moreover, anthracite modified by ZnCo-LDHs and ZnAl-LDHs had the optimal performance.

The surface characteristics and microstructures of anthracites were changed by LDH modification. The physical filter and chemical adsorption were improved after modification. The maximum adsorption capacity on ZnCo-LDH- and ZnAl-LDH-modified medium-sized anthracites were 65.79 *(mg/kg)* and 48.78 *(mg/kg)*, respectively.
